# 

**Published:** 1973-10

**Authors:** 


### BRISTOL MEDICO-CHIRURGICAL JOURNAL

OCTOBER 1973

VOL. 88 (iiii) No. 328

